# Expression of guanylyl cyclase C in tissue samples and the circulation of rectal cancer patients

**DOI:** 10.18632/oncotarget.16406

**Published:** 2017-03-21

**Authors:** Yong Liu, Guoping Cheng, Jun Qian, HaiXing Ju, YuPing Zhu, Meucci Stefano, Ulrich Keilholz, DeChuan Li

**Affiliations:** ^1^ Surgical Department of Colorectal Cancer, Zhejiang Cancer Hospital, Hangzhou, Zhejiang Province, China; ^2^ Pathology Department, Zhejiang Cancer Hospital, Hangzhou, Zhejiang Province, China; ^3^ Charité Comprehensive Cancer Center, Berlin, Germany

**Keywords:** guanylyl cyclase C, rectal cancer, tumor metastasis, disease free survival, overall survival

## Abstract

Guanylyl cyclase C (GCC) is a transmembrane surface receptor restricted to intestinal epithelial cells, from the duodenum to the rectum. We compared GCC expression in tumors and normal rectal tissues, and investigated the relation between GCC expression and metastasis and long-term survival of rectal cancer patients. Based on the UICC classification, 42 rectal cancer patients in this study were classified as stage I, 48 patients as stage II, and 90 patients as stage III. Overexpression of GCC was observed in 80 rectal tumors as compared to matched normal tissues, where no strong staining of GCC was observed. An association between GCC mRNA in the circulation and tumor emboli in vessels, CK20 mRNA, distant organ metastasis, and survival status was observed in 100 rectal cancer patients. Univariate Cox regression analysis indicated that tumor emboli in vessels, lymph node metastasis, mesenteric root lymph node metastasis and GCC mRNA correlated with 5-year disease-free survival (DFS); while lymph node metastasis, GCC mRNA, and CK20 mRNA strongly correlated with 5-year overall survival (OS). In a multivariate Cox regression model, GCC mRNA level and mesenteric root lymph node metastasis associated with DFS, while GCC mRNA levels associated with OS. Quantification of GCC expression in circulation is a valuable biomarker for assessing tumor burden and predicting outcome in rectal cancer patients.

## INTRODUCTION

Rectal cancer is one of the most common malignancies and causes of tumor-related death worldwide, with an estimated 39,220 new cases in the United States in 2016 [1, 2]. Cancer derived from the rectum is a crucial subgroup of colorectal tumors because of its location adjacent to the anus, an abundant blood supply, and the variable lymphatic drainage [2, 3]. With optimized multiple local treatment achieved by total mesorectal excision and preoperative and postoperative radiotherapy/chemoradiotherapy, local recurrence rates have been reduced. However, distant organ metastases are still the predominant causes of treatment failure in rectal cancer patients [4]. A lack of specific biomarkers for early detection of metastatic cells in circulation during local treatment or perioperative treatment is considered the main reason for metastatic treatment failure [5].

Epithelial cell adhesion molecules (EpCAMs) and cytokeratin (CK) are the essential biomarkers of colorectal cancer [5]. However, commonly used biomarkers are criticized for their reliance on cell-surface expression of epithelial-related markers, because some tumors down-regulate expression of these markers during epithelial-mesenchymal transition (EMT) [6]. Therefore, more specific and sensitive biomarkers are needed for screening and detecting colorectal cancer cells. Guanylyl cyclase C (GCC) is a transmembrane surface receptor that promotes intestinal fluid secretion, electrolyte homeostasis, and cell proliferation [7, 8]. As an important enzyme encoded by the GUCY2C gene, GCC is restricted to intestinal epithelial cells and occurs from the duodenum to the rectum but not in extra-intestinal tissues [9, 10]. GCC is a crucial tumor biomarker for the identification of occult metastases in lymph nodes and circulation when associated with prognosis of colorectal cancer (CRC) because of its highly restricted expression [11]. Comparisons of multiple epithelial cell markers (CK19, CK20, GCC, and CEA) have demonstrated that GCC is one of the most specific and sensitive markers for detecting circulating CRC cells [12–14]. GCC mRNA is an essential index for searching metastatic CRC cells in circulation, predicting tumor relapse, and predicting survival of CRC patients [12, 13, 15–17]. GCC is also an important factor in the GUCY2C hormone axis because it promotes proliferation of transit-amplifying cells in crypts, DNA damage repair, and differentiation along the secretory lineage of intestinal and epithelial cells [18–21].

Because distinctive biological behavior of rectal cancer and scarce GCC expression in rectal cancer were reported, we selected GCC as a special biomarker in tumor tissues and circulation in rectal cancer patients without distant metastasis, for designating GCC as a CRC-specific biomarker in future applications.

## RESULTS

### IHC staining of GCC in tumor tissues and normal mucosal tissues of rectum

#### Clinicopathologic characteristics of patients

The clinicopathological characteristics of patients is reported, as well as their relationship with 5-year disease-free survival (DFS) and overall survival (OS). The mean age of the patients was 55.71 years (range: 39 to 78 years), and the study population comprised 44 (55%) males and 36 (45%) females. Among these patients 15 (18.75%) were classified as stage I, 22 (27.50%) were classified as stage II, and 43 (53.75%) were classified as stage III. Among the clinicopathological characteristics, only tumor emboli in vessels show significant correlation to 5-year OS (hazard ratio [HR] = 0.163, 95% CI = 0.027 to 0.976, *P* = 0.047).

### GCC expression in tumor and normal mucosal tissues of rectum

**Figure 1:**
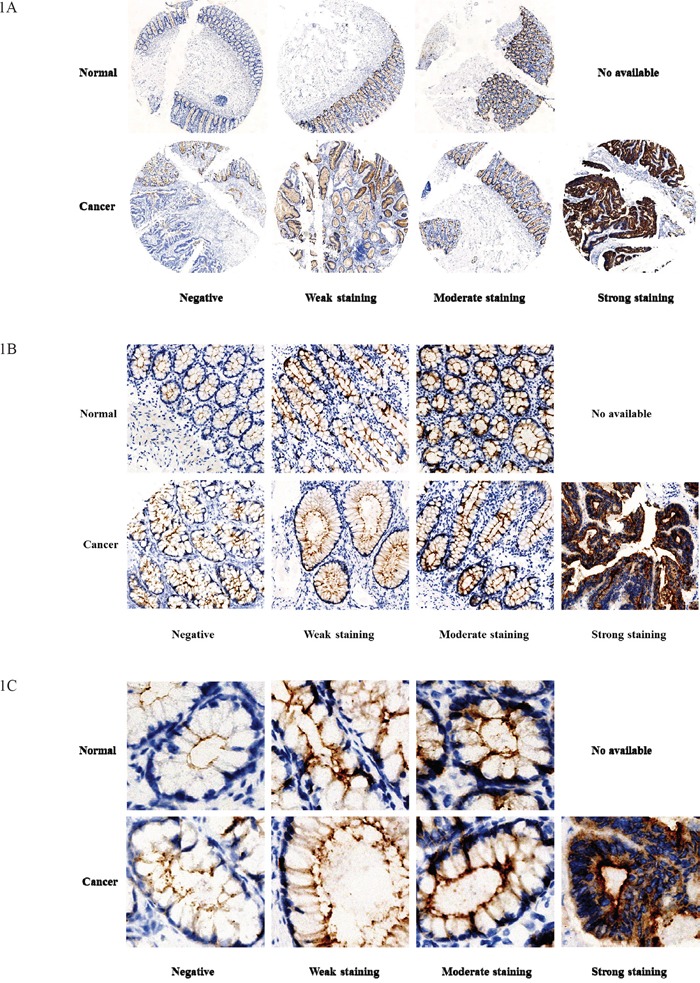
GCC expression in normal mucosal tissues and tumor tissues of rectum Negative, weak, moderate and strong staining of GCC expression in normal and tumor tissues of rectum were compared respectively in Figure 1. (**Figure 1A, 1B** and **1C**) showed GCC expression in normal and tumor tissues according to different original magnification ×20, ×100 and ×400.

GCC staining was separated into four degrees of intensity: negative, weak, moderate, and strong, to assess and compare the GCC expression in tumor and normal rectal tissues samples (Figures 1A, 1B, and 1C). Thus, rectal tumor tissue samples displayed four degrees of GCC staining, but no strong staining was observed in normal rectal tissue samples.

### Analysis of GCC staining in tumor and normal mucosal tissues of rectum

**Figure 2:**
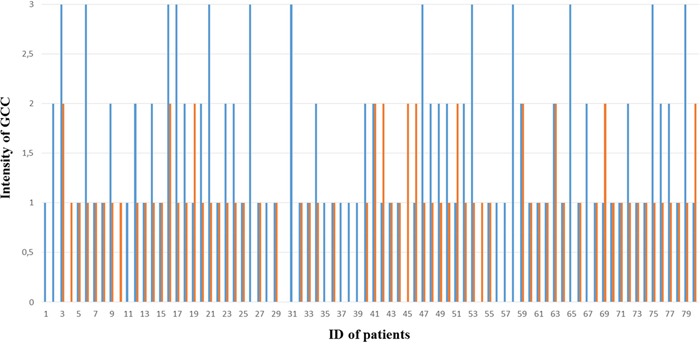
Intensity of GCC expression in 80 paired tumor and normal mucosal tissues of rectum The blue column indicates intensity of GCC expression in rectal tumor tissues, the orange column indicates intensity of GCC expression in rectal normal tissues, and no column indicates few or negative GCC expression. Serial numbers in graph were related to corresponding ID of patients.

**Figure 3:**
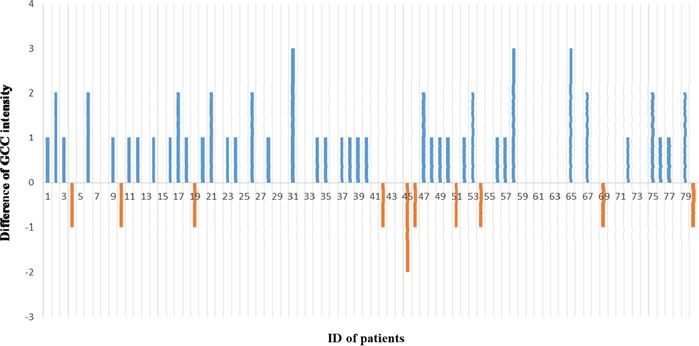
Compare of GCC intensity between paired tumor and normal tissues of rectum(tumor-normal) The blue column indicates GCC taining in tumor tissues higher than normal tissues of rectum, the orange column indicates GCC intensity in tumor tissues lower than normal mucosal tissues of rectum, and no column at baseline indicates same intensity of GCC expression between paired rectal tumor and normal tissues. Serial numbers in graph were related to corresponding patients.

As illustrated in Figure 2 and Figure 3, higher GCC expression was observed in tumor tissues than in normal mucosal tissues of the rectum, and the difference in GCC intensity highlights GCC overexpression in tumor tissues at higher frequencies than in adjacent normal tissues of the rectum. The Wilcoxon test was used to compare and assess the difference in GCC expression between tumor tissues and adjacent normal mucosal tissues of the rectum. Based on positive ranks (normal > cancer), the Z value of the Wilcoxon test was −4,352 and significant overexpression of GCC protein on tumor tissues compared with normal mucosal tissues of the rectum was observed (*P* < 0.001).

### Circulation GCC mRNA analysis of rectal cancer patients

#### Clinicopathologic characteristics of rectal cancer patients

A total of 100 rectal cancer patients was recruited for this study. The average age of the patients was 56.85 years (range: 32 to 84 years), and the study population comprised 67 (67%) males and 33 (33%) females, with tumor size > 5 cm for 31 cases (31%) and ≤ 5 cm for 69 cases (69%). Among these patients, 27 (27%) were classified as stage I, 26 (26%) were classified as stage II, and 47 (47%) were classified as stage III. Altogether, 49 patients (49%) received only surgical treatment, and 27 patients (27%) and 24 patients (24%) received additional chemotherapy and chemoradiotherapy, respectively.

### Correlation of GCC mRNA with clinical characteristics

A significant association of circulation GCC mRNA with tumor emboli in vessels (*P* = 0.005), CK20 mRNA (*P* < 0.001), distant organ metastases (*P* = 0.023), and survival status (*P* = 0.003) was observed (Table 1 and Supplementary Table 1).

**Table 1:** Correlation of GCCmRNA with clinical characteristics of rectal cancer patients

variables	n =100 (%)	CCCmRNA	
		≤500 copies/uln (%)	P value
CK20mRNA			
≤500 copies/ul	69(69%)	53(76.81%)	
>500 copies/ul	31(31%)	8(25.80%)	<0.001*
tumor emboli in vessels,			
No	73(73%)	51(69.86%)	
Yes	27(27%)	10(37.04%)	0.005*
Distal organ metastases			
No	79(79%)	53(67.09%)	
Yes	21(21%)	8(38.10%)	0.023*
Survival status			
live	89(89%)	59(66.29%)	
dead	11(11%)	2(18.18%)	0.003*

^*^ P<0.05, indicates significantly associated with subjects' demographics and clinical characteristics.

### Univariate and multivariate Cox regression model analysis

Based on univariate Cox regression model analysis, significant correlation of tumor emboli in vessels, lymph node metastases, mesenteric root lymph node metastases, and GCC mRNA with 5-year DFS, and high correlation of lymph node metastases, GCC mRNA, and CK20 mRNA with 5-year OS in rectal cancer patients were shown in our study (Table 2 and Supplementary Table 2). The variables that had a *P* value < 0.05 in univariate Cox regression analysis and those factors related to survival status and tumor metastasis were included and analyzed by use of the multivariate Cox regression model method, equivalent to Backward Stepwise (Conditional LR) analysis, to evaluate the combined effects that correlate with OS and DFS. The final analyzed variables included GCC mRNA level and mesenteric root lymph node metastasis for DFS and GCC mRNA level for OS in the multivariate Cox regression model analysis.

**Table 2:** Univariate and multivariate Cox-regression analysis of variables associated with 5 year DFS and OS

Variables	Total number	5 years DFS				5 years OS			
	(100)	Univariate		Multivariate		Univariate		Multivariate	
		N (%)	P value	OR	P value	N (%)	P value	OR	P value
tumor emboli in vessels									
No	73	11(15.06%)				5(6.85%)			
Yes	27	10(37.03%)	0.012*		0.274	6(22.22%)	0.002*		0.234
lymph node metastases									
No	54	7(12.96%)				4(7.40%)			
Yes	46	14(30.43%)	0.035*		0.197	7(15.21%)	0.203		0.503
mesenteric root lymph node metastases									
No	93	17(18.28%)				9(9.67%)			
Yes	7	4(57.14%)	0.005*	3.455	0.028*	2(28.57%)	0.064		0.400
GCCmRNA in Peripheral blood									
≤500 copy/ul	61	8(13.11%)				2(3.27%)			
>500 copy/ul	39	13(33.33%)	0.021*	2.440	0.050*	9(23.07%)	0.003*	8.147	0.008*
CK20mRNA in Peripheral blood									
≤500 copy/ul	69	12(17.39%)				4(5.79%)			
>500 copy/ul	31	9(29.03%)	0.248		0.975	7(22.58%)	0.015*		0.338

Results were represented as hazard ratio (HR) with respective 95 % confidence interval of HR (95 % CI) through univariate Cox-regression model analysis.

*P<0.05, indicates significantly associated with subjects' demographics and clinical characteristics.

## DISCUSSION

Because of its location in the pelvic cavity, surrounded by the bladder, ureter, uterus, iliac blood vessels, pelvic nerve and anterior sacral venous plexus, rectal cancer is the crucial malignancy of intestinal cancers, and it requires multiple therapies, including surgery, chemotherapy, radiotherapy, and targeted therapy [4, 22, 23]. However, because of its unique characteristics of growth, invasion, and metastasis, numerous clinical trials and research projects are designed to seek more efficacious treatment strategies for rectal cancer [23]. Besides hepatic metastasis, lung, bone, and the nervous system are the most frequently metastasized organs from rectal cancer [23, 24]. Nearly 12 cases of hepatic metastasis, 7 cases of pulmonary metastasis, 3 cases of pelvic metastasis, and 1 case of bone metastasis were observed during follow-up of our results. Significantly higher risk of lung metastasis from rectal cancer (7.5%) than from colon cancer (3.5%) among 5,230 colorectal cancer patients was reported by a multicenter study [25]. Hence, despite successful control of low local recurrence by multiple treatments, circulating and distant metastases are now the main reasons for treatment failure in early stage and advanced stage rectal cancer patients, who need precise and early detection before clinical diagnosis and treatment.

As the intestinal epithelial cell-restricted biomarker expressing only on normal, tumor, and metastatic cells from the duodenum to the rectum, GCC displayed high specificity and tissue restriction. However, the expression of the GCC receptor in normal tissue and in tumors of the rectum, as well as metastasis, still needs further investigation. Previous studies have proved that guanylin and uroguanylin are essential endogenous ligands for GCC receptors *in vivo*. Their loss and reduction at an early stage in CRC compared with matched normal adjacent tissues has been observed in both animals and humans [26, 27]. Guanylin was reportedly decreased in most colorectal tumors compared with adjacent normal mucosa [28]. Unlike paracrine hormones or ligands, GCC mRNA and protein were increased in both primary human colorectal tumors and in metastases, as compared with normal intestinal cells [29–31]. Our study showed significant higher GCC expression in tumor tissues than in normal mucosal tissues of the rectum. Similar results and conclusions were obtained by other studies, which evaluated paired samples from normal adjacent mucosa and colorectal tumors, indicating that GCC was overexpressed at the mRNA and protein levels in more than 80% of colon and rectal tumors when compared with normal adjacent intestinal mucosa [21, 30–32]. Our results indicated 50% GCC overexpression in rectal tumor tissues and 37.5% equal GCC expression and only 12.5% GCC overexpression in normal rectal tissues, which confirm the conclusions of high GCC expression in tumor tissues of the rectum. Furthermore, 13 cases of strong staining of GCC in tumor tissues but no strong staining of GCC in normal mucosal tissues of the rectum were observed in our study, which confirmed the reports of GCC overexpression in tumor tissues of colorectal cancer.

As stated earlier, distant metastasis is the main obstacle in clinical treatment of rectal cancer patients, and early detection of circulating micro-metastasis has proved to be a successful treatment and has increased long-term survival. Unlike the overexpression of GCC protein in tumor tissues from the rectum, a high GCC mRNA level in the circulation of rectal cancer patients reflects a higher circulating tumor burden in patients and correlates to distant organ metastasis and poor survival [11, 21]. To our knowledge, biomarkers such as tumor emboli in vessels and CK20 and CEA in circulation always correlate to treatment failure and poor survival of patients [13, 33, 34]. Tumor emboli in vessels is the common pathologic factor for a tumor cluster in the drawing vessels from a tumor location, and it is also the pathological proof of circulating micro-metastasis [33]. The association of tumor emboli in vessels with poor survival, tumor recurrence, and tumor diversion were widely reported and proved to be highly significant [35–38]. Our studies indicated significant correlation of tumor emboli in vessels with both DFS and OS, and a highly significant association of GCC mRNA in circulation with tumor emboli in vessels, CK20 mRNA, distant organ metastases, and survival status was further proved. Studies revealed that tumors with blood vessel invasion and lymph node metastasis were significantly associated with poor prognosis in CRC patients in multivariate analysis [38, 39]. The same was confirmed by our study. Furthermore, together with mesenteric root lymph node metastases, GCC mRNA was included in a multivariate Cox regression model analysis of DFS and OS, which indicated high a predictive value of GCC mRNA levels in survival analysis of rectal cancer patients. The presence of mesenteric root lymph node metastases indicated a high frequency of hematogenous metastasis and advanced clinical stage of patients, and it correlated with poor OS, even though standard chemotherapy and radiotherapy were performed after radical surgery [40–42]. The incidence of metastasis to the mesenteric root lymph node was reported to be relatively low in CRC patients, ranging from 0.3% to 11.1% [40, 41], but their 5-year survival rates were significantly lower as opposed to patients without lymph node metastasis [41]. In our study, mesenteric root lymph node metastases showed high correlation with high GCC mRNA level as well as poor DFS and OS, which greatly enhanced the effect of GCC mRNA in the assessment of CRC cell spreading and the patients' survival predictions.

Our study revealed a higher GCC expression in tumor tissues than in normal tissues of the rectum and a significant correlation of high GCC mRNA in circulation with tumor emboli in vessels, CK20 mRNA, distal organ metastasis, and poor survival, which may promote the clinical application of GCC as a survival predictor for assessing tumor burden and a valuable biomarker for guiding treatment strategies in the future.

## MATERIALS AND METHODS

### Patient information

All patients had surgical treatment at the Surgical Department of Colorectal Cancer of the Zhejiang Cancer Hospital, Hangzhou, China. We recruited 80 patients with rectal cancer for tumor tissue immunohistochemistry (IHC) analysis and 100 patients with rectal carcinomas for circulating GCC mRNA and CK20 mRNA detection. Periphery blood from 5 healthy donors without colorectal cancer was included as negative controls. Patients with a known second neoplastic disease or benign intestinal tumors were excluded for our studies, and stage IV rectal cancer cases and patients who received chemoradiotherapy before surgical treatment were excluded from GCC analysis. Routine pathological examinations were applied to all 260 tumor samples from the 180 patients. Based on the Union for International Cancer Control Classification of Colorectal Cancer, stage I to stage III rectal cancer patients were included. Stage II and III patients at risk for metastases were treated with standard venous or oral chemotherapy regimens after surgery. The follow-up was performed at periodic intervals with CT scan, tumor biomarker detection, and colonoscopy, as well as by letter, telephone, and self-comprehensive review, to make sure that patients were alive and to evaluate whether they had developed local recurrences or distant organ metastases. All study protocols were approved by the Institutional Review Board, and informed consent was obtained from all study participants.

### IHC staining of GCC in rectal cancer tissue

Paired tumor tissues and adjacent normal tissues of the rectum from 80 patients were sliced and gradually dewaxed with concentrated ethanol, followed by a 5-minute blocking step to prevent endogenous peroxidase activity. Immunostaining was conducted by use of mouse anti-human GC-C monoclonal antibody (537) (Santa Cruz Bio, Dilution: 1: 200). Slides were incubated for 1 hour at room temperature (37°C). After PBS washing, HRP-labeled goat anti-mouse EnVision Flex secondary antibody (Dako Diagnostics, Glostrup, Denmark; 1: 100 dilution) was applied to slides for 20 minutes. After another PBS washing, slides were incubated for 5 minutes in DAB solution (DAKO Diagnostics, Glostrup, Denmark) until cell membrane staining was observed by microscope, washed in deionized water, counterstained with hematoxylin, and dehydrated with xylene before mounting. All the tissue specimens were observed and evaluated by two pathologists.

### Circulating mRNA detection from rectal cancer patients

Blood samples were drawn simultaneously for the detection of GCC mRNA and CK20 mRNA levels. Peripheral venous blood was obtained at the time of clinical staging before surgery. The first 2 mL of blood was discarded to minimize the possibility of false-positives, and 5 mL of blood was then collected into EDTA-containing vacutainer tubes. All samples were processed within 2 hours of collection, immediately stored in cryovials, frozen in liquid nitrogen, and stored at −80°C until further processing. GCC mRNA and CK20 mRNA detected by quantitative real-time RT-PCR were extracted from peripheral blood by use of Trizol (Invitrogen, USA), per the manufacturer's instructions. Primer design, PCR amplification, and mRNA detection were performed as previously described [11]. CEA and CA199 were detected by use of an ARCHITECT i2000 kit (Abbott Diagnostics; Abbott Park, IL, USA) for routine enzyme immunoassays.

### Assessment of IHC staining and scoring

Only cells exhibiting distinct apical membrane staining were considered positive for GCC expression. The semi-quantitative score system and assessment was used to generate an overall score for each set of tissue samples. Based on this approach, an overall staining index (score values 0-12) was determined by multiplying the staining intensity score by the positive percentage per visible area under microscope score. The staining intensity was scored as follows: 0, negative; 1, weak; 2, moderate; and 3, strong. The frequency of positive cells was defined as follows: 0, less than 5%; 1, 6%-25%; 2, 26%-50%; 3, 51%-75%; and 4, 75%-100%. Overall scores were then divided as indexes into four categories as follows: negative (score 0), weak (score 1-4), moderate (score 5-8), and strong (score 9-12).

### Statistic assessment

Chi-square tests were performed to determine the relation of the IHC staining score by GCC antibody and GCC mRNA to other clinical and pathological characteristics. Further IHC staining of GCC between rectal cancer and normal tissues were analyzed by rank sum test based on overall scores of GCC staining. We selected a cutoff value of 500 copies for GCC mRNA and CK20 mRNA based on the manufacturer's instructions and previous results [43]. The mRNA copy numbers and clinicopathologic characteristics were assessed with DFS and OS. The characteristics that showed significant association with DFS and OS in univariate analysis were put into a multivariate Cox regression model analysis equivalent to Backward Stepwise (Conditional LR) analysis. Multivariate analysis was performed to estimate the HR for survival per mRNA copy numbers adjusted by other characteristics. Kaplan-Meier curves with log-rank tests were also performed to evaluate OS or DFS for a given mRNA level and clinicopathologic variables. All statistical tests were two-sided and had a 95% CI. *P* < 0.05 was considered statistically significant. All statistical analyses were performed by PASW Statistics Software, Version 23.0 (SPSS Inc, Chicago, IL, USA).

## SUPPLEMENTARY MATERIALS FIGURES AND TABLES




