# Supplementary material for: Expression of guanylyl cyclase C in tissue samples and the circulation of rectal cancer patients
Source: Oncotarget. 2017 Mar 21;8(24):38841–9. doi: 10.18632/oncotarget.16406 (PMC5503576; doi:10.18632/oncotarget.16406)
Supplement: Supplementary file 1 [file oncotarget-08-38841-s001.pdf]

Expression of guanylyl cyclase C in tissue samples and the circulation of rectal cancer patients

SUPPLEMENTRY TABLES

Supplementary Table 1: Correlation of GCCmRNA level with other clinical characteristics of rectal patients

See Supplementary File 1

Supplementary Table 2: Univariate and multivariate Cox-regression analysis of factors associated with 5 year DFS and OS

See Supplementary File 1
