# Supplementary material for: Expression of guanylyl cyclase C in tissue samples and the circulation of rectal cancer patients
Source: Oncotarget. 2017 Mar 21;8(24):38841–9. doi: 10.18632/oncotarget.16406 (PMC5503576; doi:10.18632/oncotarget.16406)
Supplement: Supplementary file 2 [file oncotarget-08-38841-s002.doc]

Table 1 Correlation of GCCmRNA level with other clinical characteristics of rectal patients

| variables | n =100 (%) | CCCmRNA | |
| --- | --- | --- | --- |
|  |  | >500 copies/ul  n (% ) | P value |
| age |  |  |  |
| ≤50 years | 29(29%) | 17(58.62%) |  |
| >50 years | 71(71%) | 44(61.97%) | 0.755 |
| sex |  |  |  |
| Females | 33(33%) | 21(63.63%) |  |
| Males | 67(67%) | 40(59.70%) | 0.704 |
| tumor size |  |  |  |
| Less than 5 cm | 69(69%) | 42(60.86%) |  |
| More than 5 cm | 31(31%) | 19(61.29%) | 0.968 |
| Pathological type |  |  |  |
| Ulcerative type | 28(28%) | 16(57.14%) |  |
| Protruded | 72(72%) | 45(62.50%) | 0.622 |
| differentiation type |  |  |  |
| Well | 86(86%) | 51(59.30%) |  |
| Poor | 14(14%) | 10(71.42%) | 0.388 |
| Infiltration depth |  |  |  |
| Mucosal and muscular infiltrating | 37(37%) | 24(64.86%) |  |
| Serosal invasion | 63(63%) | 37(58.73%) | 0.544 |
| tumor emboli in vessels, |  |  |  |
| No | 73(73%) | 51(69.86%) |  |
| Yes | 27(27%) | 10(37.04%) | 0.005* |
| lymph node metastases |  |  |  |
| No | 54(54%) | 36(66.67%) |  |
| Yes | 46(46%) | 25(54.34%) | 0.208 |
| mesenteric root lymph node metastases |  |  |  |
| No | 93(93%) | 58(62.36%) |  |
| Yes | 7(7%) | 3(42.85%) | 0.307 |
| TNM stage |  |  |  |
| Stage I | 27(27%) | 17(62.96%) |  |
| Stage II | 26(26%) | 18(69.23%) |  |
| Stage III | 47(47%) | 26(55.32%) | 0.491 |
| Postoperative chemotherapy |  |  |  |
| No | 49(49%) | 30(61.22%) |  |
| Chemotherapy | 35(35%) | 19(54.28%) |  |
| Oral chemotherapy | 16(16%) | 12(75.00%) | 0.371 |
| CEA |  |  |  |
| ≤5 ng/ml | 61(61%) | 39(63.93%) |  |
| >5 ng/ml | 39(39%) | 22(56.41%) | 0.452 |
| CA199 |  |  |  |
| ≤37 U/ml | 81(81%) | 48(59.26%) |  |
| >37 U/ml | 19(19%) | 13(68.42%) | 0.461 |
| CK20mRNA |  |  |  |
| ≤500 copies/ul | 69(69%) | 53(76.81%) |  |
| >500 copies/ul | 31(31%) | 8(25.80%) | <0.001* |

Subjects’ demographics and clinical characteristics were represented as n (%). Dispersion of GCC mRNA level were summarized as n (%) for a given subjects’ demographics and clinical characteristics and compared using a non-parametric method, Mann–Whitney U test or Kruskall Wallis test, due to the ordinal data type of GCC mRNA level.

*P<0.05, indicates significantly associated with subjects’ demographics and clinical characteristics.

Table 2 Univariate and multivariate Cox-regression analysis of factors associated with 5 year DFS and OS

| Variables | N  (100) | 5 years DFS | | | | 5 years OS | | | |
| --- | --- | --- | --- | --- | --- | --- | --- | --- | --- |
|  |  | Univariate | | Multivariate | | Univariate | | Multivariate | |
|  |  | N (%) | P value | OR | P value | N (%) | P value | OR | P value |
| age |  |  |  |  |  |  |  |  |  |
| ≤50 years | 29 | 7(24.13%) |  |  |  | 3(10.34%) |  |  |  |
| >50 years | 71 | 14(19.72%) | 0.726 |  |  | 8(11.26%) | 0.824 |  |  |
| sex |  |  |  |  |  |  |  |  |  |
| Females | 33 | 7(21.21%) |  |  |  | 3(9.09%) |  |  |  |
| Males | 67 | 14(20.89%) | 0.977 |  |  | 8(11.94%) | 0.648 |  |  |
| tumor size |  |  |  |  |  |  |  |  |  |
| ≤ 5 cm | 69 | 15(21.73%) |  |  |  | 6(8.69%) |  |  |  |
| > 5 cm | 31 | 6(19.35%) | 0.794 |  |  | 5(16.13%) | 0.288 |  |  |
| Pathological type |  |  |  |  |  |  |  |  |  |
| Ulcerative type | 28 | 8(28.57%) |  |  |  | 3(10.71%) |  |  |  |
| Protruded | 72 | 13(18.05%) | 0.270 |  | 0.503 | 8(11.11%) | 0.992 |  | 0.792 |
| differentiation type |  |  |  |  |  |  |  |  |  |
| Well | 86 | 18(20.93%) |  |  |  | 9(10.46%) |  |  |  |
| Poor | 14 | 3(21.43%) | 0.952 |  | 0.805 | 2(14.28%) | 0.597 |  | 0.284 |
| Infiltration depth |  |  |  |  |  |  |  |  |  |
| Mucosal and muscular infiltrating | 37 | 6(16.21%) |  |  |  | 3(8.10%) |  |  |  |
| Serosal invasion | 63 | 15(23.81%) | 0.399 |  | 0.713 | 8(12.69%) | 0.468 |  | 0.562 |
| tumor emboli in vessels |  |  |  |  |  |  |  |  |  |
| No | 73 | 11(15.06%) |  |  |  | 5(6.85%) |  |  |  |
| Yes | 27 | 10(37.03%) | 0.012* |  | 0.274 | 6(22.22%) | 0.002* |  | 0.234 |
| lymph node metastases |  |  |  |  |  |  |  |  |  |
| No | 54 | 7(12.96%) |  |  |  | 4(7.40%) |  |  |  |
| Yes | 46 | 14(30.43%) | 0.035* |  | 0.197 | 7(15.21%) | 0.203 |  | 0.503 |
| mesenteric root lymph node metastases |  |  |  |  |  |  |  |  |  |
| No | 93 | 17(18.28%) |  |  |  | 9(9.67%) |  |  |  |
| Yes | 7 | 4(57.14%) | 0.005* | 3.455 | 0.028* | 2(28.57%) | 0.064 |  | 0.400 |
| TNM stage |  |  |  |  |  |  |  |  |  |
| Stage I | 27 | 4(14.81%) |  |  |  | 2(7.40%) |  |  |  |
| Stage II | 26 | 3(11.53%) |  |  |  | 2(7.69%) |  |  |  |
| Stage III | 47 | 14(29.78%) | 0.097 |  | 0.316 | 7(14.89%) | 0.494 |  | 0.524 |
| CA199 values in Peripheral blood |  |  |  |  |  |  |  |  |  |
| ≤37U/ml | 81 | 14(17.28%) |  |  |  | 7(8.64%) |  |  |  |
| >37U/ml | 19 | 7(36.84%) | 0.063 |  | 0.109 | 4(21.05%) | 0.110 |  | 0.059 |
| CEA values in Peripheral blood |  |  |  |  |  |  |  |  |  |
| ≤5 ng/ml | 61 | 9(14.75%) |  |  |  | 5(8.19%) |  |  |  |
| >5 ng/ml | 39 | 12(30.77%) | 0.060 |  | 0.224 | 6(15.38%) | 0.186 |  | 0.280 |
| Postoperative chemotherapy |  |  |  |  |  |  |  |  |  |
| No | 49 | 8(16.32%) |  |  |  | 6(12.24%) |  |  |  |
| Chemotherapy | 35 | 11(31.42%) |  |  |  | 4(11.42%) |  |  |  |
| Oral chemotherapy | 16 | 2(12.50%) | 0.763 |  | 0.788 | 1(6.25%) | 0.795 |  | 0.618 |
| GCCmRNA in Peripheral blood |  |  |  |  |  |  |  |  |  |
| ≤500 copy/ul | 61 | 8(13.11%) |  |  |  | 2(3.27%) |  |  |  |
| >500 copy/ul | 39 | 13(33.33%) | 0.021* | 2.440 | 0.050* | 9(23.07%) | 0.003* | 8.147 | 0.008* |
| CK20mRNA in Peripheral blood |  |  |  |  |  |  |  |  |  |
| ≤500 copy/ul | 69 | 12(17.39%) |  |  |  | 4(5.79%) |  |  |  |
| >500 copy/ul | 31 | 9(29.03%) | 0.248 |  | 0.975 | 7(22.58%) | 0.015* |  | 0.338 |

Results were represented as hazard ratio (HR) with respective 95 % confidence interval of HR (95 % CI) through univariate Cox-regression model analysis

*P<0.05, indicates significantly associated with subjects’ demographics and clinical characteristics
